# K-13 propeller gene polymorphisms isolated between 2014 and 2017 from Cameroonian *Plasmodium falciparum* malaria patients

**DOI:** 10.1371/journal.pone.0221895

**Published:** 2019-09-03

**Authors:** Carole Else Eboumbou Moukoko, Fang Huang, Sandrine Eveline Nsango, Loic Pradel Kojom Foko, Serge Bruno Ebong, Patricia Epee Eboumbou, He Yan, Livia Sitchueng, Bouba Garke, Lawrence Ayong

**Affiliations:** 1 Biological Sciences Department, Faculty of Medicine and Pharmaceutical Sciences, University of Douala, Douala, Cameroon; 2 Malaria Research Unit, Centre Pasteur Cameroon, Yaoundé, Cameroon; 3 Malarial Department, National Institute of Parasitic Diseases, Chinese Center for Disease Control and Prevention, Shanghai, China; 4 Parasitology and Entomology Research Unit, Department of Animal Organisms, Faculty of Science, University of Douala, Douala, Cameroon; 5 Animal Organisms Biology and Physiology Department, Faculty of Sciences, University of Douala, Douala, Cameroon; 6 Bonassama Hospital, Douala, Cameroon; 7 Centre Pasteur Cameroon, Garoua Center, Yaoundé, Cameroon; Kyung Hee University, REPUBLIC OF KOREA

## Abstract

The emergence of artemisinin-resistant parasites since the late 2000s at the border of Cambodia and Thailand poses serious threats to malaria control globally, particularly in Africa which bears the highest malaria transmission burden. This study aimed to obtain reliable data on the current state of the kelch13 molecular marker for artemisinin resistance in *Plasmodium falciparum* in Cameroon. DNA was extracted from the dried blood spots collected from epidemiologically distinct endemic areas in the Center, Littoral and North regions of Cameroon. Nested PCR products from the Kelch13-propeller gene were sequenced and analyzed on an ABI 3730XL automatic sequencer. Of 219 dried blood spots, 175 were sequenced successfully. We identified six K13 mutations in 2.9% (5/175) of samples, including 2 non-synonymous, the V589I allele had been reported in Africa already and one new allele E612K had not been reported yet. These two non-synonymous mutations were uniquely found in parasites from the Littoral region. One sample showed two synonymous mutations within the kelch13 gene. We also observed two infected samples with mixed K13 mutant and K13 wild-type infection. Taken together, our data suggested the circulation of the non-synonymous K13 mutations in Cameroon. Albeit no mutations known to be associated with parasite clearance delays in the study population, there is need for continuous surveillance for earlier detection of resistance as long as ACTs are used and scaled up in the community.

## Introduction

Between 2000 and 2016, the global malaria incidence and mortality due to *Plasmodium falciparum* decreased by 18% with the largest reductions recorded in Southeast Asia, Latin America, and Africa following the introduction of artemisinin-based combination therapies (ACTs) [1,2]. ACTs are adopted as the first-line combination treatment of uncomplicated malaria and have become the cornerstone of malaria treatment all over the world [1,3].

The therapeutic efficacy of ACTs is threatened by an unusual resistant phenotype that manifests as delayed clearance of *P*. *falciparum* blood forms following artemisinin-based treatment. Such resistance phenotypes first appeared in western Cambodia, before spreading to the Greater Mekong Subregion, Southeast Asia and then Southern China [4–15]. This greatly impeded malaria control efforts in the area particularly due to lack of similarly effective replacement therapies [16]. To improve antimalarial resistance monitoring, molecular markers of artemisinin resistance have been identified to occur within the *P*. *falciparum* kelch (K13)-propeller gene, and in total, more than 200 different K13 alleles have been reported in Southeast Asia [9, 17, 18]. Based on *in vitro* and *in vivo* studies, 13 non-synonymous mutations occurring within the K13 propeller domain have been shown to be a major determinant of artemisinin resistance [9, 11, 13, 16–19]. Although the K13 mutation C580Y has been identified as the most strongly associated mutation with resistance phenotype against artemisinin, there are five other validated *K13* mutants (N458Y, Y493H, R539T, I543T, R561H) [16].

Major concerns exist on the possibility of emergency and/or spreading of artemisinin resistance to African countries as previously reported for chloroquine and sulfadoxine-pyrimethamine [20–22]. In African countries, different K13 gene mutations were reported but non-synonymous mutations are still rare and highly diverse [23–34]. Furthermore, the point mutations were unrelated to K13 polymorphisms found to be associated with reduced susceptibility in Asia [16, 29, 30]. Different studies have also reported an association between severe pediatric malaria cases and recurrent infections with various drug resistance associated polymorphisms-[23, 35, 36]. The situation would be dramatically difficult to face if artemisinin resistance was formally reported in Africa as the continent accounts for the bulk of the malaria burden. The scenario is not far from reality given the existence of some conditions and common practices in African populations, such as self-medication with antimalarial drugs or counterfeit/fake drugs [37–39], which can increase the risk of emergence of artemisinin resistant strains.

Since 2014, Cameroon has adopted ACTs artesunate-amodiaquine (ASAQ) and artemether–lumefantrine (AL) as first-line treatment of uncomplicated malaria [40]. A few past studies on K13 polymorphism in Cameroon reported no evidence of K13 mutations associated with artemisinin resistance [29, 32–34, 41, 42]. However, this previous studies were limited to the Centre and South West Regions of Cameroon with distinct epidemiological profiles when compared to most other regions of the country and, described eight non-synonymous mutations with great diversity [29, 32–34, 41, 42]. This study aimed to investigate the level of polymorphism of K13 gene of *P*. *falciparum* isolates from three epidemiologically distinct regions of Cameroon to assess the artemisinin-based treatment failure in Cameroon from the perspective of parasite genetics. The molecular markers of artemisinin resistance are therefore critical to assess the distribution of K13 polymorphism, and have the potential to support disease surveillance systems to provide data that can alert the emergence or spreading of *P*. *falciparum* mutants [23].

## Materials and methods

### i) Study design and population

A prospective hospital-based study was conducted between 2014 and 2017 in the context of the Centre Pasteur Cameroon’s routine antimalarial drug resistance surveillance program.

**Fig 1:**
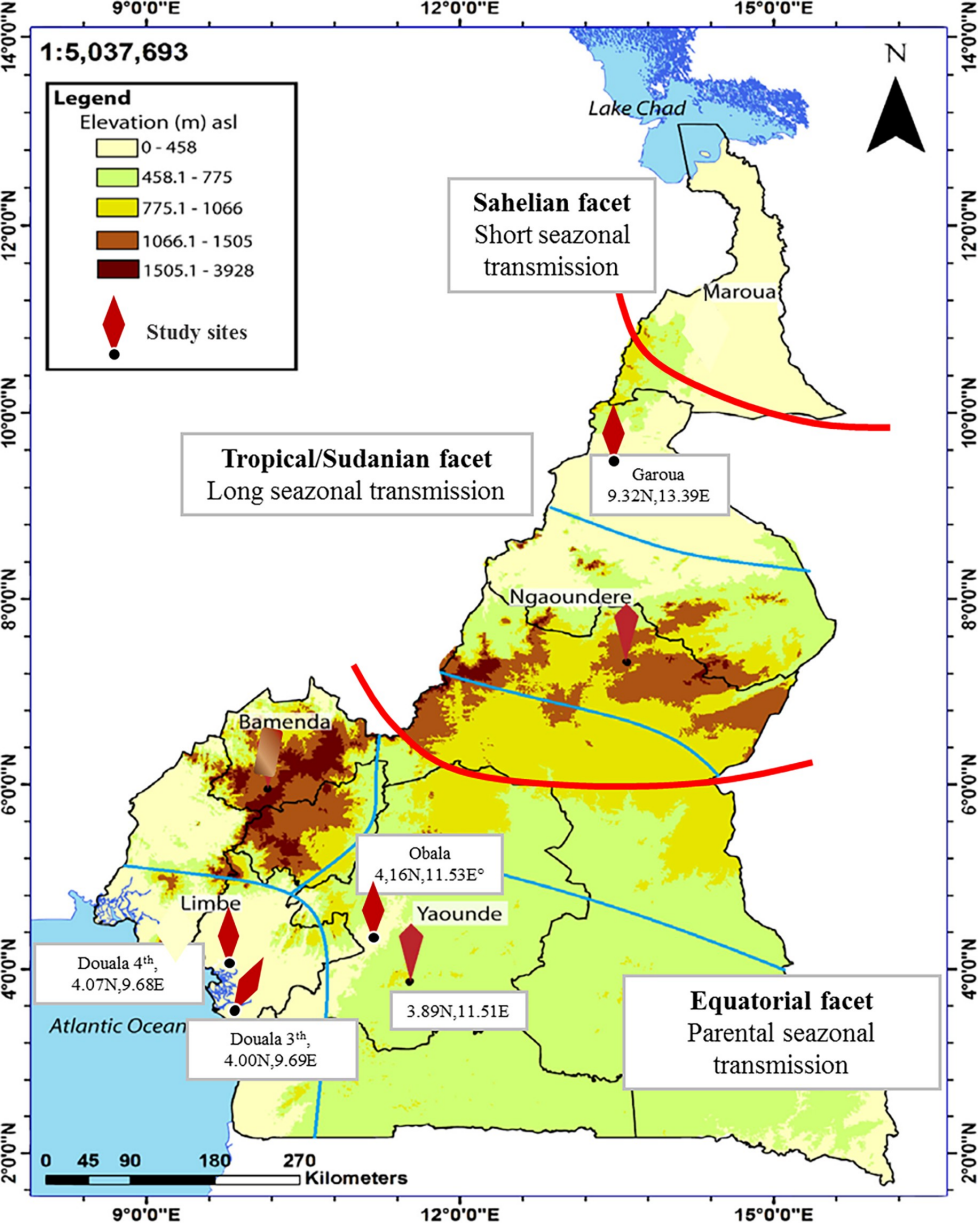
Map depicting the study sites selected. Five epidemiological strata and 3 major epidemiological facets are delineated.

The study was carried out in two epidemiological facets (Equatorial and Tropical/Sudanian facets) of Cameroon (Fig 1).

The Equatorial facet included two transmission settings in the Littoral region (Douala 3^th^ and 4^th^ district), two settings in the Centre region (Yaoundé and Obala). The Tropical/Sudanian facet included one setting (Garoua) in the North Region. According to the Mapping Malaria Risk in Africa (MARA) epidemiological stratification, Cameroon is categorized from South to North under 3 major epidemiological facets: (i) the Equatorial facet that is characterized by large forests and dense vegetation with extensive hydrographic network, hot and humid climate, heavy rainfall (5000 mm per year), and perennial transmission of malaria parasites, (ii) the Tropical/Sudanian facet that include the North Region, characterized by savannas, steppes, shrubs and gallery forests, and marked by long (4–6 months) and intense seasonal (rainy season) transmission of malaria parasites, and (iii) the Sahelian facet in the Far North zone characterized by hot and dry tropical climate where malaria transmission is short seasonal (1 to 3 months). Some areas within the North region of the country (Far North and some Northern Health Districts) and some high altitude areas exhibit epidemic potential [43]. The entomological inoculation rates vary from 100 infective bites per man and per month in the Equatorial facet to about 10 infective bites per man per month in both the Tropical/Sudanian and Sahelian facets [43].

Samples were collected post-ACT period in 2014 at the North region, in 2016 in the Littoral region and between 2016 and 2017 in the Centre region.

In the absence of reliable national data on the prevalence of point mutations in Cameroon, we used data sources from one previous study conducted on *P*. *falciparum* K13-propeller polymorphisms on samples collected in November 2012 to March 2013 from Yaoundé and Douala, it was determined that a minimal sample size of 50 samples per site would be needed [33]. We used a convenience non-probability sampling applicable in the study when the members of the population are convenient to sample. To limit the selection and information biases, participants were enrolled consecutively and participation in the study was voluntary.

The survey’s target population was all febrile patients consulting at the selected health facilities and local residents and, all of target population screened by rapid diagnostic test (RDT) for presence of malaria parasites. Eligibility for inclusion was defined as all febrile local residents, with only *P*. *falciparum* parasite infection as confirmed by thick and thin blood smears microscopy, and absence of recent prescription or antimalarial self-medication within the 15 days’ period prior to enrolment, and who had not travelled out of the study site within the last 3 weeks. All patients with signs and symptoms of severe or complicated malaria and pregnant women were excluded.

The questionnaire was administered independently the same day by two persons (a PhD student-interviewer-1 and a master student-interviewer-2) interviewing the patients within 5min intervals, about their use of antimalarials within the 15 days’ period prior to enrolment to estimate inter-interviewer reproducibility. Patients did not receive any information sheets on antimalarials during these two interviews to minimize the risk of answer change. Data was collected using structured questionnaires to collect information on the socio-demographic characteristics of the study population.

Information sheets on antimalarial drugs and antimalarial drugs resistance were provided to all patients after the interview. Completing the questionnaire emphasis was placed on the importance of a confirmed diagnostic test before the use of antimalarial drugs treatment, as a tool in infection control that can promote better self-management. This sheet allowed to have information on what are the antimalarial drugs? what is antimalarial drugs resistance? What is "inappropriate" use of antimalarial drugs and what can physicians, other health professionals and the public do to fight gain the abuse of antimalarial drugs consumption and resistance.

This study was conducted in accordance with ethics directives related to research on humans in Cameroon. The study was approved by the Cameroonian Ministry of Public Health and, administrative authorization was obtained from all the health facilities. Before enrollment and the administration of questionnaire, subjects were informed on the purpose and process of the investigation (background, goals, methodology, study constraints, data confidentiality, and rights to opt out from the study), and a signed informed consent was obtained from all those who agreed to participate in the study in accordance with the Helsinki Declaration. Participation was voluntary, anonymous and without compensation. All patients were treated in accordance to the treatment guidelines from the Cameroon National Malaria Control Program.

### ii) Blood collection and parasite density

RDT, thick and thin blood smear were prepared from collected finger-pick blood samples before treatment of patients. Blood samples were also spotted on filter papers and stored at 4°C until use for DNA extraction and molecular analysis. Parasite densities were determined by thick blood smear microscopy with quality-control by a World Health Organization (WHO) certified microscopist [44]. The *P*. *falciparum* asexual stages were counted against 500 white blood cells (WBCs), and parasite densities expressed as the number of asexual parasites per micro liter (μl) using the following formula: Parasite density = Number of parasites x 8000/Number of WBC, where 8000 is the assumed number of WBC per μl of blood.

### iii) Molecular amplification and sequencing

Parasites genomic DNA was extracted from dried blood spots using the QIAamp 96 DNA Blood Kit (Qiagen, Valencia, CA) according to the manufacturer’s instructions. DNA was eluted with 100 μl TE (Tris-HCl 10mM, EDTA 0.5mM, pH 9.0) buffer (Qiagen, Valencia, CA) and stored at -20°C until use.

A nested PCR amplification method (Takara PCR kit) of the K13-propeller domain (>440 amino acids) codon was used following previously reported protocols [17] with minor modifications. Briefly, PCR products were purified using filter plates (Edge Biosystems, Gaithersburg, MD) and directly sequenced and analyzed on an ABI 3730XL automatic sequencer as recommended by the manufacturer. Primer sequences for the K13-propeller domain PCR amplification, sequencing as well as the cycling conditions are presented in Table 1.

**Table 1:** Primers sequences, nested PCR and sequencing conditions.

Primer sequences	Activities	Procedure conditions	Size of PCR products
K1-F: 5’- cggagtgaccaaatctggga-3’ K4-R: 5’- gggaatctggtggtaacagc-3’	Nested PCR	95°C×2 min, 30 cycles [95°C×30 sec, 60°C×90 sec, 72°C × 90 sec], 72°C ×10 min	2097 bp
K2-F: 5’- gccaagctgccattcatttg -3’ K3-R: 5’-gccttgttgaaagaagcaga-3’		95°C×2 min, 30 cycles [95°C×30 sec, 60°C×90 sec, 72°C × 90 sec], 72°C ×10 min	850 bp
K2-F: 5’- gccaagctgccattcatttg -3’ K3-R: 5’-gccttgttgaaagaagcaga-3’ K5-F: 5’-ttatgtcattggtggaactaa-3’ K6-R: 5’-tctaggggtattcaaaggtgc-3’	Sequencing	ABI 3730XL automatic sequencer	

The amplification products were analyzed by 1.5% agarose gel electrophoresis before sequencing. Purified products were sequenced by an ABI 3730XL automatic sequencer and sequence data were analyzed using the Genome Assembly Program GAP 4 to identify any variation across the gene that could result in a non-synonymous or synonymous Single Nucleotide Polymorphisms (SNPs). Bi-directional sequencing was used and all the products had been sequenced twice using independently amplified PCR products. The successful amplified sequences were analyzed using the Basic Local Alignment Search Tool (BLAST) to compare nucleotide sequences from samples to the reference genome PF3D7_1343700, and then individual alleles were identified for each locus allowing identification of the amino acid residues at the SNP. All mutant samples were independently checked to ensure that all the mutants were real mutant.

### iv) Data analysis

Categorical variables were expressed as frequencies, whereas numerical variables (Age) were presented as means and 95% confidence intervals (CI), as data followed a normal distribution. All continuous variables which did not respect the normality hypothesis were log-transformed.

## Results

### Characteristics of *P*. *falciparum* infected patients in population survey

A total of 219 Cameroonian *P*. *falciparum* isolates were collected from 4 sites, 50, 50, 70 and 49 samples from Yaoundé, Douala 3^th^ district, Douala 4^th^ district and Garoua respectively. The majority of the patients was males (52.8%) and 75% reported, had fever in the previous 48h as shown in Table 2. One hundred and sixty-nine patients (169) of the 219 samples were obtained from patients who attended hospitals and, the remain was from the community.

**Table 2:** Basic characteristics of *P*. *falciparum* infected patients according to different epidemiological areas of Cameroon.

		Equatorial facet			Tropical/Sudanian
	All areas	Douala 3^th^ district	Douala 4^th^ district	Yaoundé	Garoua
Sample size	219	50 (22.8)	70 (32.0)	50 (22.8)	49 (22.4)
Male:Female ratio	52.8:47.2	38.0:62.0	64.3:35.7	55.1:44.9	49.0:51.0
Mean age (95%CI)*, years	5.5 (4.8–6.2)	7.1 (7.8–8.5)	4.7 (3.6–5.7)	4.5 (3.4–5.6)	6.0 (3.6–8.3)
Geometric mean of parasite density [Range], parasites/μl	14,004 [40–551,56]	5633 [960–36,000]	4644 [40–551,56]	3351 [174–464,096]	40,166 [6,320–91,000]

Data are number (%) otherwise indicated.

*, 95%CI: Confidence interval.

The mean age was 6.3 years and most of the patients were less than 5 years old. Overall, no significant difference was observed in terms of mean age between men and women (p = 0.973) in our study population.

Geometric mean parasite density was 14,004 parasites/μL with a IQ(InterQuartile)_25-75_: 4,560–45,800 parasites/μL. The parasitaemia were significantly highest in the North region (40,165 parasites/μL, IQ_25-75_: 40,000–45,800 parasites/μL, p = 0.001) and Centre region (32,241 parasites/μL, IQ_25-75_: 13,776–145,888 parasites/μL, p = 0.0001) compared to Littoral region. The mean parasitaemia of patients from Littoral region was 5,109 parasites/μL, IQ_25-75_: 1,600–16,000 parasites/μL.

### K13-propeller sequence polymorphisms and national distribution

Sequences of K13-propeller domain were generated successfully for 175 (79.9%) isolates. Most samples without successful sequencing were from the Douala 4 district. 44 samples could not be interpreted because of poor quality of the dried blood spots or an insufficient quantity of DNA.

Nearly all samples (169/175, 96.6%) contained a wild-type allele. We identified six SNPs (6/175, 3.4%) in 5 isolates, including 4 synonymous (4/175, 2.3%) and 2 non-synonymous (2/175, 1.1%) SNPs in the study population subjects (Table 3). The 4 synonymous (66.7%) K13 mutations were G449G, G453G, C469C and G625G. The two (33.3%) non-synonymous mutations of the samples with K13 mutations were V589I and E612K, which were found in two samples from Douala in the 3th and 4^th^ health districts. The K13 non-synonymous mutation on the codon 589 was identified in a female patient aged 3 years and having parasite density of 9,600 parasites/μL blood while, the K13 non-synonymous mutations on the codon 612 was isolated in a male patient aged 14 years and having parasite density of 7,360 parasites/μL blood. The only sample from the Yaoundé site with K13 mutation presented with two synonymous mutations on codon 449 and 453. Of the two mixed K13 wild-type and mutant infections observed, one was a non-synonymous mutation on codon 589 whereas the second was synonymous on codon 625 (Table 3).

**Table 3:** Synonymous and non-synonymous single-nucleotide polymorphisms in K13-propeller gene in Cameroon.

Sample ID	No of Codon	Wild-type allele		Mutant allele		Type of mutation	Infection
		Sequence (nt)	AA	Sequence (nt)	AA		
YdeNkol0166	449	ggT	Gly	ggG	Gly	G449G Synonymous	Single
YdeNkol0166	453	ggT	Gly	ggG	Gly	G453G Synonymous	Single
Gar05003	469	tgC	Cys	tgT	Cys	C469C Synonymous	Single
DlaEbong185	589	Gtc	Val	Atc/Gtc	Iso	V589I Non synonymous	Wild-type + mutant
DlaBona056	612	Gaa	Glu	Aaa	Lys	E612K Non synonymous	Single
DlaBona099	625	ggA	Gly	ggG/ggA	Gly	G625G Synonymous	Wild-type + mutant

AA: amino acid; nt: nucleotide; Gly(G) = Glycin; Cys(C) = Cystein; Glu(E) = Glutamic acid; Val(V) = Valine; Iso(I) = Isoleucine; Lys(K) = Lysine. Capital letter in the codon sequence indicates the muted nucleotide

## Discussion

With the continued pressure for natural selection of the parasites due to widespread adoption and persistent use of ACTs as first line antimalarial treatments in Africa, there are rising fears of artemisinin resistance emergence across the continent which may impede the control efforts in these countries.

In Cameroon, the ACTs including Artesunate-Amodiaquine (ASAQ) and Artemether-Lumefantrine (AL) were adopted in 2004 and 2006 respectively, for the treatment of uncomplicated malaria. Its use has increased significantly following the nationwide implementation in the health facilities [45]. Therefore, drug pressure due to an uncontrolled use (prescription or self-medication) of ACTs and artesunate monotherapy might have selected resistant parasites over time.

We aimed in this study to determine the frequency of SNPs within the K13 propeller sequence of circulating *P*. *falciparum* parasites as a means of assessing the risk of emerging artemisinin resistance among local parasite populations. Indeed, the discovery and validation of *P*. *falciparum* K13 SNPs linked to artemisinin resistance in Southeast Asia [9,14,19,46], supports the continuous targeting of the K13 propeller gene in surveillance efforts worldwide to provide tools for public health systems to deliver effective interventions.

Our findings showed the absence of all previously characterized artemisinin resistance-associated SNPs reported in Southeast Asia, from sequencing analyses of 219 *P*. *falciparum* isolates representing a cross section of parasite populations in Cameroon. These findings are supported by recent molecular epidemiologic data currently showing no molecular evidence for artemisinin resistance (*in vivo* or *in vitro* studies) in sub-Saharan Africa including one study we conducted in Cameroon [16,17,30–33,41,47–50]. Overall findings are encouraging and suggest that artemisinin resistance is not yet established in Africa and in Cameroon.

The present data reported also a very low prevalence (3.4%) of non-synonymous K13 mutations in the *P*. *falciparum* isolates and we found diversity in K13-propeller sequence. This is consistent with previous reports conducted in Cameroon. To date, seven epidemiological-molecular studies had been conducted in Cameroon which showed a heterogeneity of mutations in the parasite population 10 years after the implementation of ACTs in the Centre and South West regions of the country [29,32–34,41,42,51]. These studies indicated that less than 4% of all samples showed a mutation in the K13 gene and none were among those associated with artemisinin clearance delay in Southeast Asia. In Central, West and East Africa, the allelic frequencies reported were generally rare less than 6% [24,27,34,36,49,52,53]. However, one study in Cameroon revealed a high rate, 15.1% of isolates presented at least one non-synonymous SNP in K13 gene [42].

Furthermore, the most frequent non-synonymous polymorphisms (A578S) observed in Africa [11,16,25–27,29,30,49,52] which we also recently reported from a sample collected in 2013 from asymptomatic patients in Yaoundé [33] was not detected in the cohort. The closeness of this allele with C580Y associated with delayed parasite clearance in Southeast Asia and with tolerance to artemisinin *in vitro* [16,17], and its possible ability to affect the tertiary structure of the K13 protein thus modify the function of the protein suggested by a computational modeling [54] had led to the belief that it would have a potential role in the prolonged clearance under artesunate treatment observed in a study conducted among 78 children with severe malaria in Uganda [36,38]. But recent studies have suggested that this allele is not an artemisinin-resistance mutation [11,16,25,36].

In a recent study we reported SNPs among 15/590 samples collected in Yaoundé and Douala in 2012–2013 with four non-synonymous mutations (Y482S, A569S, A578S and F583S) only in Yaoundé [33]. Whereas SNPs were not observed in the three studies conducted among 251, 11 and 22 samples collected in 2015 in another health facility in Yaoundé, Central region of Cameroon, in 2013–2014 in Buea, West region of Cameroon in 2013–2014, and between 2012–2015 among migrant workers who returned to Henan Province from Africa, respectively [29, 32, 51]. In the other studies, high level of diversity was found, such as K189T mutation which was high prevalent among samples from local residents in two studies conducted in rural and semi-urban areas of the South West Region (24.3%-58/239 and et 42.4%-14/33) [41,42]. Some non-synonymous SNPs (K189T, K189N and N217K) found in samples from this part of Cameroun had previously been described [11,24,25,30].

The non-synonymous V589I mutation widely distributed as it has been reported in others African countries such as Mali and Madagascar [11,13,30,32,55] was found with a wild-type infection in one sample from Douala. This could be due to the translation of gene flux between settings. The phenotype conferred by this mutation is still unknown, although no study has yet reported its involvement in the resistance to artemisinin [16].

We report in this study, a novel mutant variant E612K in simple infection in one isolate from Douala. No genetic similarities were found to mutant parasites described elsewhere. It would be interesting to continue characterizing the clinical significance of these two mutations in artemisinin resistance in Africa and Ring stage assays (RSA_0-3h_) can adequately allow for the validation of K13 mutant as a resistance marker to artemisinin [56]. Thus, adequate evaluation of whole individuals (asymptomatic persons and symptomatic patients) *in vivo* and *in vitro* studies are needed to determine the potential implications of this mutation or other new molecular markers in artemisinin sensitivity. This is supported by the recent study which reported a persistence of parasitaemia on Day 3 among 9 Senegalese patients with wild-type for K13 allele [50].

Taken together, the low selected frequencies of k13 mutant alleles found in Cameroon suggests ART resistant parasites are not under evolutionary selection in Cameroon, therefore reinforcing the assumption that such mutations are rare in Africa. Furthermore, none of the polymorphisms known to be involved in artemisinin resistance in Asia were really associated in artemisinin resistance in Africa. Thus, local artemisinin resistant *P*. *falciparum* strains may emerge independently in Cameroon and in the African continent under ACTs constant drug pressure, misuse of ACTs, self-medication with antimalarial drugs, use of counterfeit drugs adding to the intense transmission [37,38,57].

Unlike other studies conducted in Cameroon and elsewhere, the different study sites are geographically distant; this does not influence the possible detection of site-specific mutations as a geographic proximity of study areas at a single time point which may limit the ability to detect differences in the molecular profiles of drug resistance among the areas [58]. The lack of RSA data, clinical evaluation or *vitro* assessment that would have added more pertinent information on the susceptibility level of this new mutant to ACT is a major limitation. The other limit is the fact that only patients with uncomplicated malaria were enrolled which may have limited the diversity of the parasite population analyzed.

## Conclusion

Our data suggest that under intense malaria transmission and use of ACTs in Cameroon from 2006, K13 mutations have not been selected in Douala, Yaoundé, and Garoua. Non-synonymous K13 mutations are still rare and highly diverse. Only two non-synonymous K13 mutations have been reported in this study with one newly described mutation (E612K). The validation of the K13 mutant as a resistance marker requires it to be correlated with slow clearance in clinical studies, reduced drug sensitivity in *ex-vivo* assays or *in vitro* assays (e.g., RSA 0-3h) and continuing molecular surveillance of artemisinin resistance.

## Abbreviations

ACTs: artemisinin-based combination therapies
ASAQ: artesunate-amodiaquine
AL: artemether–lumefantrine
BVGH: BIO Ventures for Global Health
IQ25-IQ-75: Second and fourth InterQuartile (IQ) above the median
ISRT-Health: International Society for Health Research and Training
MARA: Mapping Malaria Risk in Africa
RDT: rapid diagnostic test
SD: standard deviation
WHO: World Health Organization
